# Beyond Powerhouses: Roles of Mitochondria, from Development to Therapeutic Potential

**DOI:** 10.3390/biology14101367

**Published:** 2025-10-07

**Authors:** Susana P. Pereira, Ludgero C. Tavares

**Affiliations:** 1 UCIBIO—Applied Molecular Biosciences Unit, Department of Chemistry, NOVA School of Science and Technology, Universidade NOVA de Lisboa, 2829-516 Caparica, Portugal; 2 Associate Laboratory i4HB—Institute for Health and Bioeconomy, NOVA School of Science and Technology, Universidade NOVA de Lisboa, 2829-516 Caparica, Portugal; 3 CIVG—Vasco da Gama Research Center, University School Vasco da Gama—EUVG, 3020-210 Coimbra, Portugal

Mitochondria, long recognized as the powerhouse of the cell, have emerged as master regulators of cellular fate and therapeutic potential through a broad range of signaling pathways and metabolic modulation. This Special Issue of Biology brings together ten original research and review articles that collectively trace a compelling arc, from the role of mitochondria in reproduction and early-life programming to their dysfunction in pathophysiology and emergence as novel therapeutic targets.

This Special Issue begins by focusing on the reproductive lifespan, as mitochondrial quality control is essential in fertility and gamete viability. Costa et al. [1] contribute a comprehensive review of mitochondrial quality control mechanisms in male fertility, highlighting how disruptions in mitophagy, biogenesis, and dynamics can impair spermatogenesis and sperm function. Their work underlines the importance of mitochondrial surveillance systems in maintaining reproductive health and suggests that mitochondrial dysfunction may underlie idiopathic male infertility.

Complementing this topic, Moniz et al. [2] explore the impact of cryopreservation and transplantation on mitochondrial function in gonadal grafts. Employing a rodent model, they demonstrate that both procedures significantly compromise mitochondrial membrane potential and respiratory capacity, contributing to the low survivability of transplanted tissue. Their findings emphasize the need for improved preservation protocols that safeguard mitochondrial integrity to enhance reproductive outcomes.

Bracchi et al. [3] extend the reproductive theme by investigating the cardiometabolic consequences of Rebaudioside A exposure during the reproductive stage. Their experimental research in female rats reveals that a chronic intake of this non-caloric sweetener alters mitochondrial function in cardiac tissue, leading to increased oxidative stress and impaired energy metabolism. These results raise important questions about the long-term metabolic effects of dietary additives during sensitive reproductive windows.

In the Guest Editor’s experience [4,5,6,7,8,9,10,11,12,13,14,15,16], a large body of research has focused on how the maternal environment shifts early-life mitochondrial programming, exerting lasting effects on offspring health. In this Special Issue, this theme is further explored; Lomas-Soria et al. [17] show that maternal obesity induces premature aging in mitochondrial electron transport chain genes in the liver of rat offspring, with sex-specific differences. Their transcriptomic analysis reveals the downregulation of key mitochondrial genes in male offspring, suggesting that maternal metabolic status can epigenetically reprogram mitochondrial function in a sex-dependent manner.

Similarly, Yan et al. [18] report that maternal nutrient excess reduces the number of mitochondria and activates stress signaling in fetal baboon skeletal muscle. Using a non-human primate model, they demonstrate that overnutrition during pregnancy leads to mitochondrial depletion and increased expression of unfolded protein response markers, potentially predisposing offspring to metabolic dysfunction. These findings reinforce the concept that mitochondrial health is shaped in utero and may influence disease susceptibility later in life.

Metabolic programming is currently a research hotspot, and as we learn more, increasing attention is being given to the mitochondrial contribution to the onset of pathophysiological processes and the progression of chronic diseases. Mitochondrial dysfunction can not only reflect underlying metabolic disturbances but also actively contribute to pathophysiological mechanisms across various organ systems. For instance, Kulovic-Sissawo et al. [19] investigated mitochondrial dysfunction in endothelial progenitor cells (EPCs), revealing that EPCs exhibit impaired mitochondrial respiration and increased oxidative stress compared to mature endothelial cells. Their study suggests that mitochondrial deficits in EPCs may compromise vascular repair capacity, with implications for cardiovascular disease and aging, highlighting a potential mechanistic link between mitochondrial dysfunction and cardiovascular aging.

Similarly, Amorim et al. [20] explore the progression of non-alcoholic fatty liver disease (NAFLD/MASLD) to hepatocellular carcinoma, as a paradigm of maladaptive mitochondrial responses. Their review demonstrates how impaired β-oxidation, excess ROS production, and disrupted mitochondrial dynamics drive a cascade from steatosis to fibrosis and ultimately to malignancy, positioning mitochondria as both biomarkers and active players in liver disease progression.

Extending this perspective to genetic mitochondrial disorders, Tomczewski et al. [21] provide a phenotypic characterization of female mice heterozygous for Tafazzin deletion, a model of Barth syndrome. They report subtle but significant alterations in cardiac and skeletal muscle mitochondrial function, even in the absence of overt pathology. This study underscores the importance of considering carrier status and sex-specific effects in mitochondrial diseases, and it adds to our understanding of how partial gene deletions can manifest subclinically.

Together, these studies illustrate that mitochondrial dysfunction, whether developmentally programmed, environmentally induced, or genetically inherited, serves as a critical nexus in disease pathogenesis and warrants further investigation as both a target and indicator in the context of aging and chronic disease progression.

Thus, the final arc of this Special Issue explores therapeutic strategies targeting mitochondrial dysfunction. Chang et al. [22] demonstrate that Formoterol, a β2-adrenoreceptor agonist, restores mitochondrial function in cells harboring a Parkinson’s disease-related UQCRC1 mutation. Their data show improved mitochondrial dynamics, transport, and bioenergetics, suggesting that β2-agonists may offer neuroprotective benefits by modulating mitochondrial homeostasis.

Closing this Special Issue, Lin et al. [23] explore the bidirectional relationship between mitochondria and the complement system. Their review synthesizes evidence that mitochondrial damage can activate complement pathways while complement proteins can localize to mitochondria and influence their function. This “double-edged sword” highlights the complex interplay between innate immunity and mitochondrial signaling in health and disease.

Collectively, these ten manuscripts offer a panoramic view of mitochondrial biology, from its foundational role in reproduction and development to its critical involvement in disease and its potential as a therapeutic target. Far from being mere energy factories, mitochondria emerge here as dynamic regulators of cellular fate, often exhibiting sex-specific idiosyncrasies. As our mechanistic understanding deepens, so too does the potential of mitochondria-centered strategies to diagnose, treat, and prevent a wide array of complex diseases.

The Guest Editors would like to gratefully acknowledge the Biology Editorial Office for their continued support, as well as the peer reviewers, authors, and respective research units for their valuable contributions. We hope that this Special Issue will provide both insight and inspiration, to enable continued development in this rapidly advancing field.
